# Supplementary figures and images for: The novel role of LDHA/LDHB in the prognostic value and tumor-immune infiltration in clear cell renal cell carcinoma
Source: PeerJ. 2023 Aug 1;11:e15749. doi: 10.7717/peerj.15749 (PMC10402698; doi:10.7717/peerj.15749)

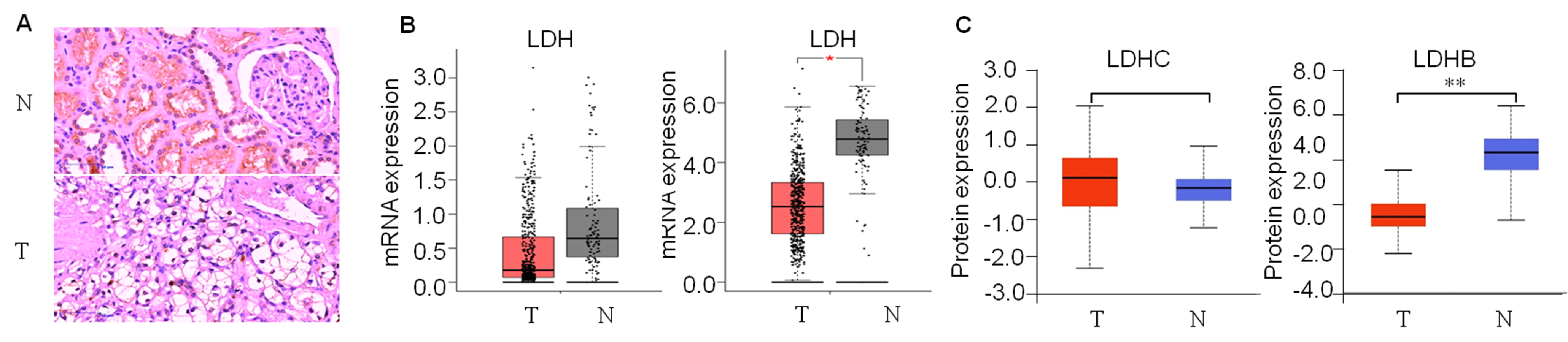

Supplement: Figure S1 — T: ccRCC, N: normal kidney tissues. *: P < 0.05, **: P < 0.01. [file peerj-11-15749-s001.png]

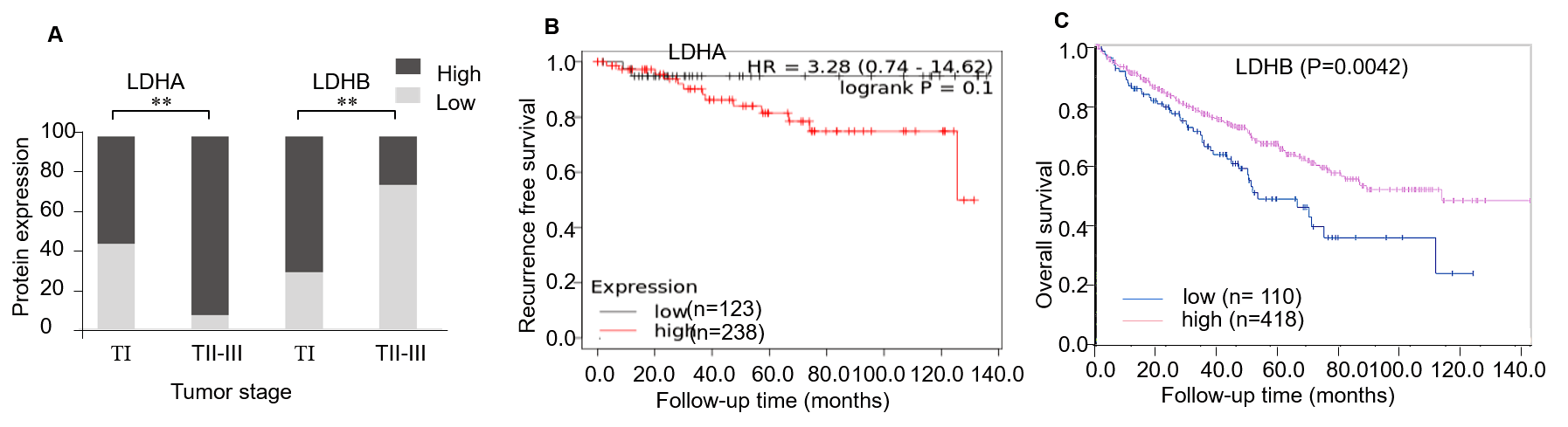

Supplement: Figure S2 — A: The relationship between LDHA/LDHB and ccRCC stage (IHC). B: The recurrence-free survival (RFS) of LDHA in 530 ccRCC patients (K–M plotter). C: The overall survival (OS) of LDHB in 528 ccRCC patients (HPA). [file peerj-11-15749-s002.png]

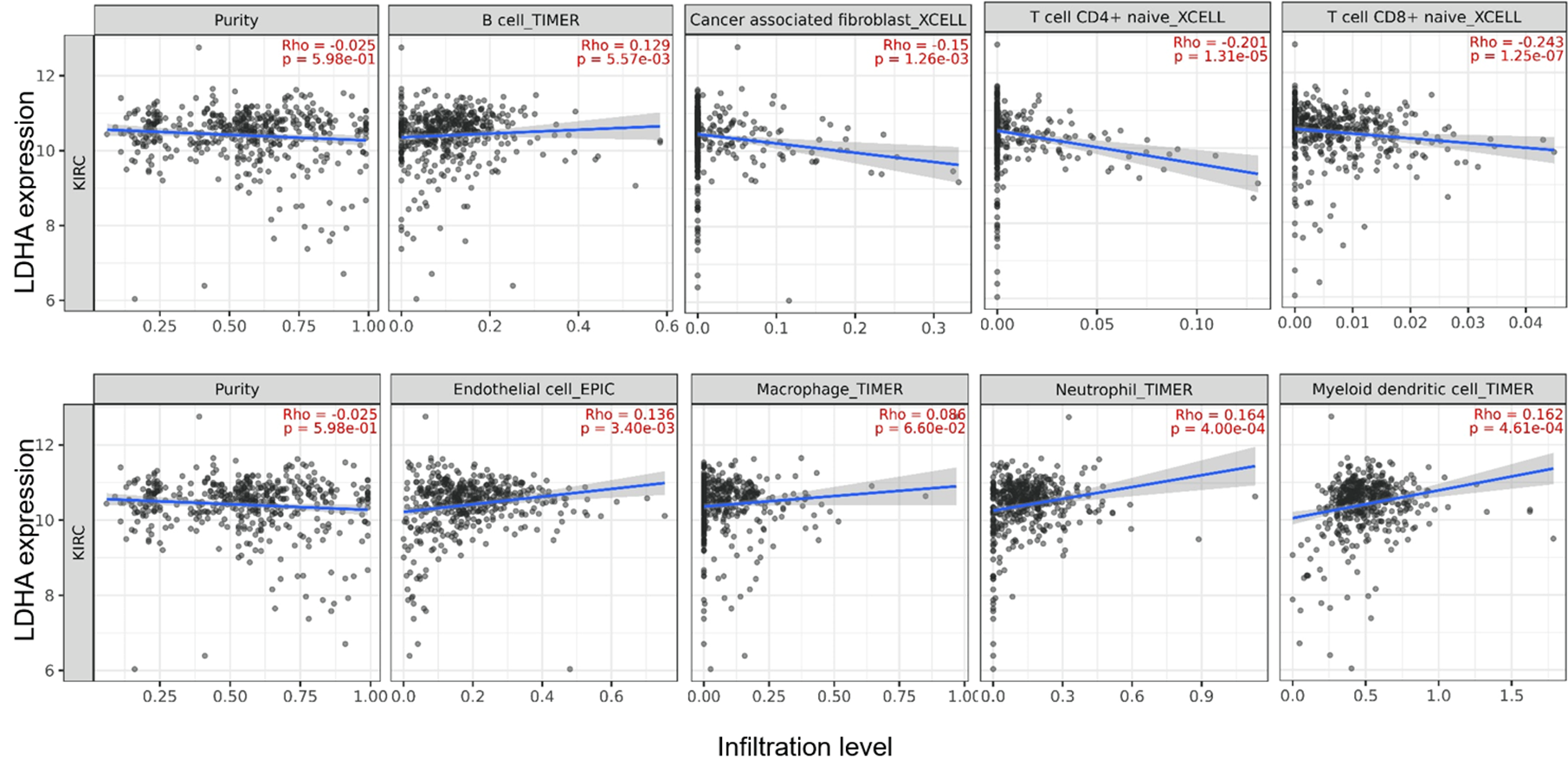

Supplement: Figure S3 — The infiltration levels of the eight TIIC subsets, i.e., B cell (TIMER), Cancer associated fibroblast (XCELL), CD4 + T cell (XCELL), CD8 + T cell (XCELL), Endothelial cell (EPIC), Macrophage (TIMER), Myeloid dendritic cell (TIMER) and Neutrophil (TIMER). [file peerj-11-15749-s003.png]

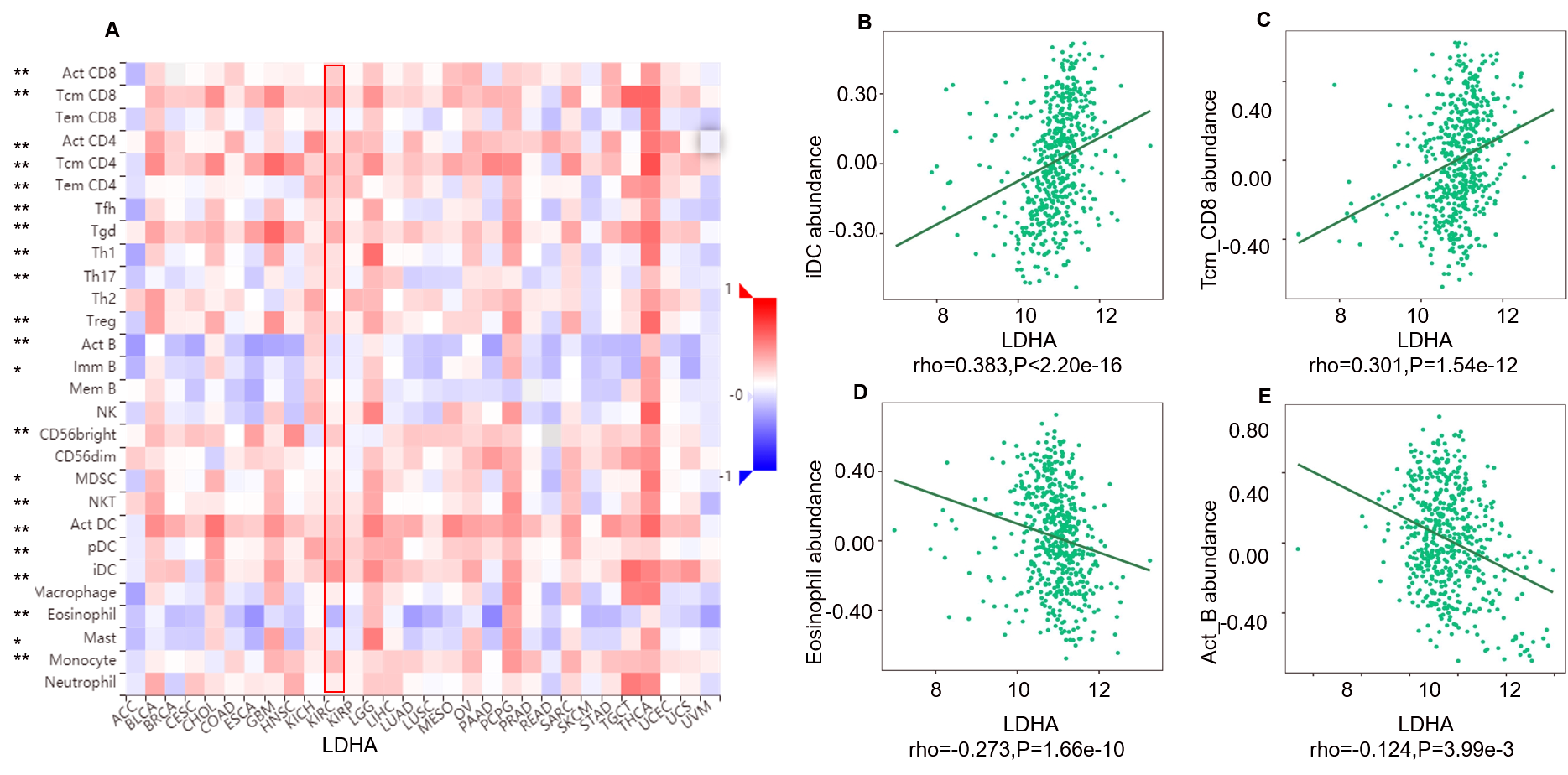

Supplement: Figure S4 — A: The pan-cancer analysis of relationship between LDHA expression and abundance of the 28 tumor-infiltrating lymphocytes (TILs). The top four lymphocytes either positive (B: iDC cell, C: Tcm_CD8 cell) or negative (D: Eosinophil cell, E: Act_B) correlation with LDHA expression in ccRCC patients. *P < 0.05, **P < 0.01. [file peerj-11-15749-s004.png]

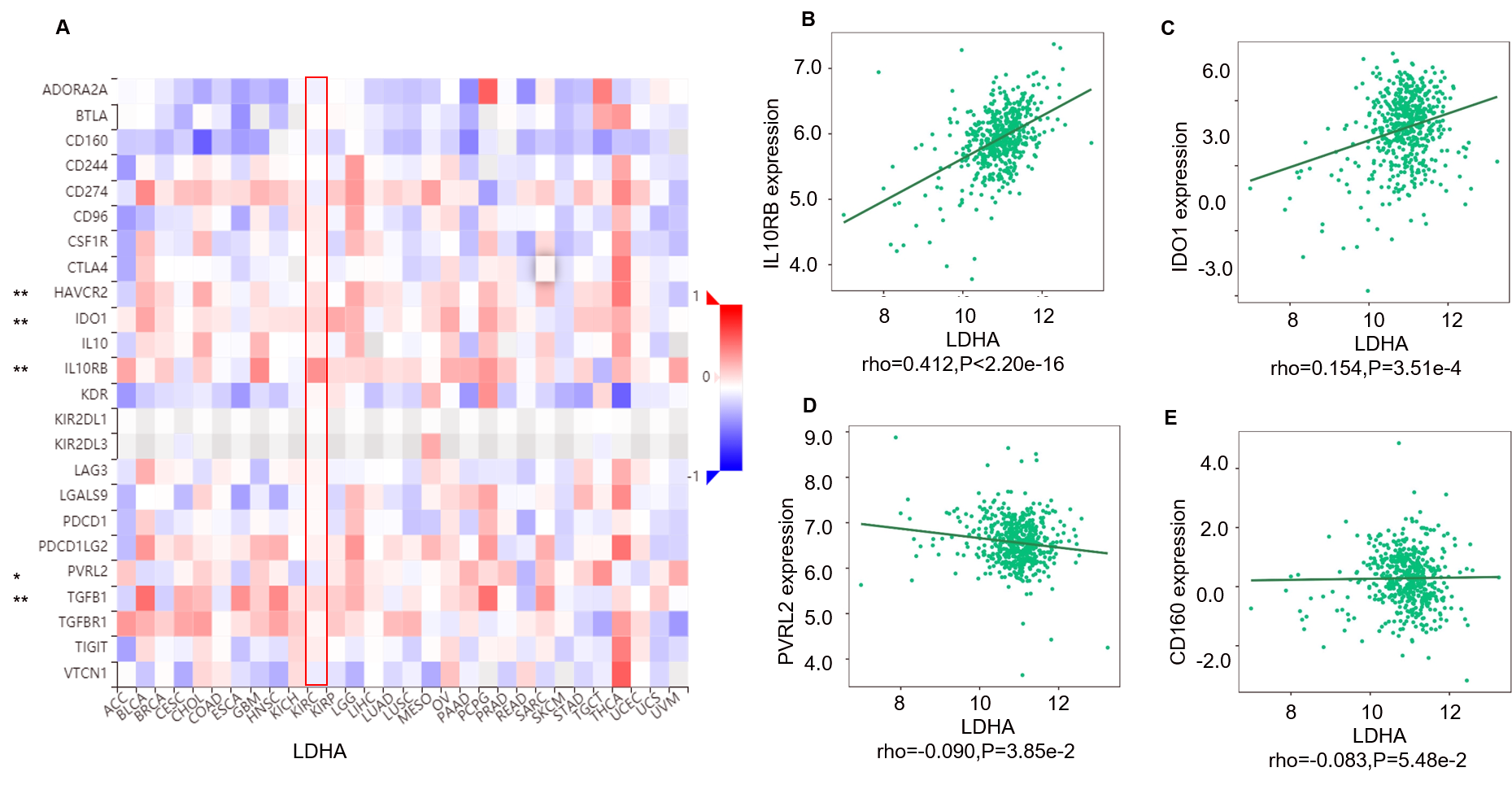

Supplement: Figure S5 — A: The pan-cancer analysis of relationship between LDHA expression and abundance of the 24 immunoinhibitors. The top four immunoinhibitors [IL10RB (B), IDO1 (C), PVRL (D) and CD160 (E)] either positively or negatively correlated with LDHA expression in ccRCC patients. *P < 0.05, **P < 0.01. [file peerj-11-15749-s005.png]

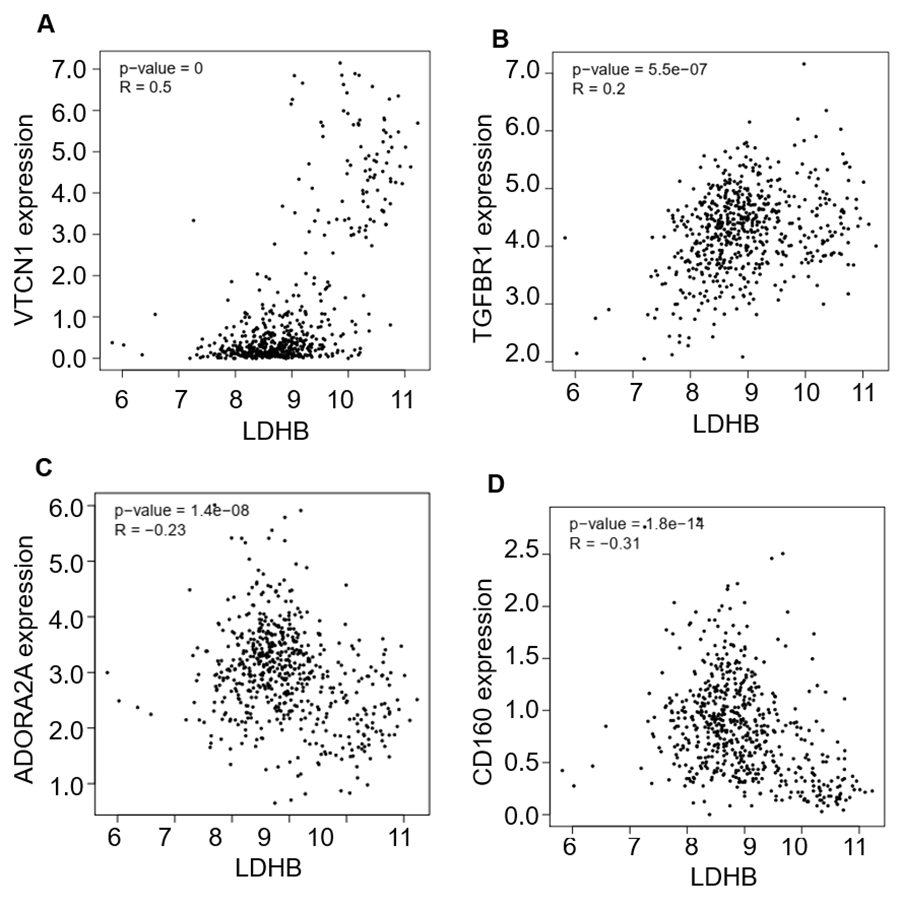

Supplement: Figure S6 [file peerj-11-15749-s006.png]
