# Supplementary material for: The novel role of LDHA/LDHB in the prognostic value and tumor-immune infiltration in clear cell renal cell carcinoma
Source: PeerJ. 2023 Aug 1;11:e15749. doi: 10.7717/peerj.15749 (PMC10402698; doi:10.7717/peerj.15749)
Supplement: Data S2 [file peerj-11-15749-s008.docx]

**The novel role of LDHA/LDHB in the prognostic value and tumor-immune infiltration in clear cell renal cell carcinoma**

Jie Chen^1^, Fei Wu^2,3^, Yehua Cao^4^, Yuanxin Xing^5^, Qingyong Liu^3*^, Zuohui Zhao^6*^

^1^ Department of Urology, Jinan Central Hospital, Cheeloo College of Medicine, Shandong University, Jinan, Shandong, China

^2^ Department of Urology, Shandong Provincial Hospital Affiliated to Shandong First Medical University, Shandong First Medical University &Shandong Academy of Medical Sciences, Jinan, Shandong, China.

^3^ Department of Urology, The First Affiliated Hospital of Shandong First Medical University & Shandong Provincial Qianfoshan Hospital, Jinan, Shandong, China

^4^ Department of Gastroenterology, Jinan Central Hospital, Cheeloo College of Medicine, Shandong University, Jinan, Shandong, China

^5^ Central Laboratory, Jinan Central Hospital, Cheeloo College of Medicine, Shandong University, Jinan, Shandong, China

^6^ Department of Pediatric Surgery, The First Affiliated Hospital of Shandong First Medical University & Shandong Provincial Qianfoshan Hospital, Jinan, Shandong, China

Corresponding Author:

Qingyong Liu^1^

Jingshi Road, No. 16766, Jinan, Shandong, 250014, China

Email address: [lqylbc@163.com](mailto:lqylbc@163.com)

Zuohui Zhao^2^

Email address: zhaozuohui@126.com, zhaozuohui@sdhospital.com.cn

**Supplementary dataset file 2: Original full-length gel/blot bands**

| The original full-length gel/blot bands | The marked full-length gel/blot bands |
| --- | --- |
| 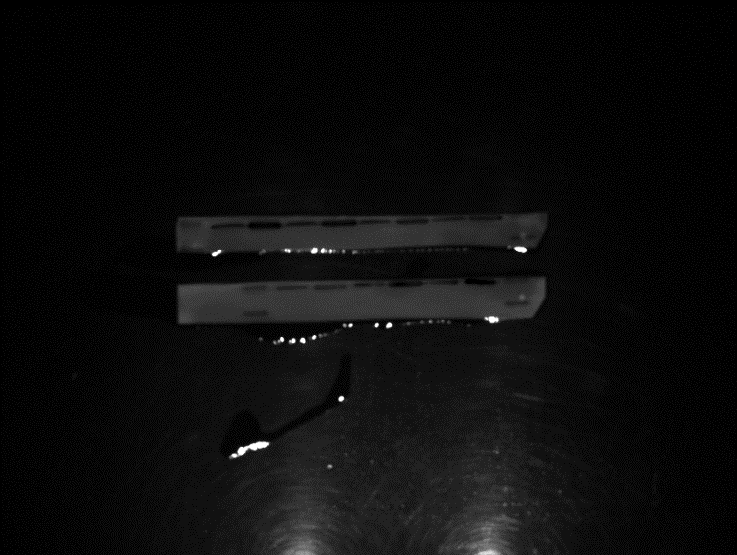 | 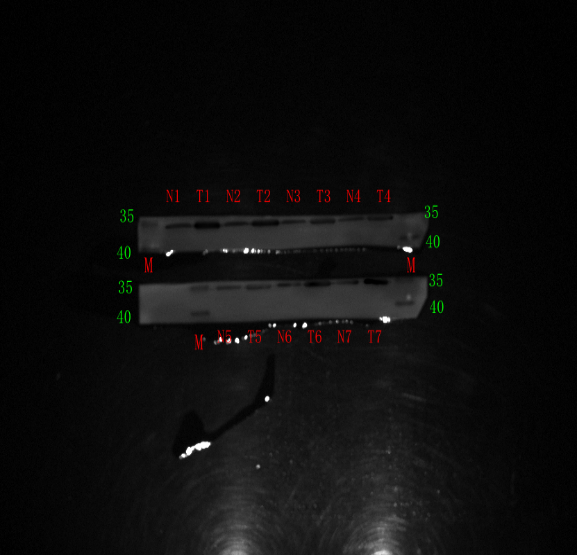 |

1. The original full-length gel/blot bands of LDHA in 7 pairs of ccRCC and adjacent tissues.

| The original full-length gel/blot bands | The marked full-length gel/blot bands |
| --- | --- |
| 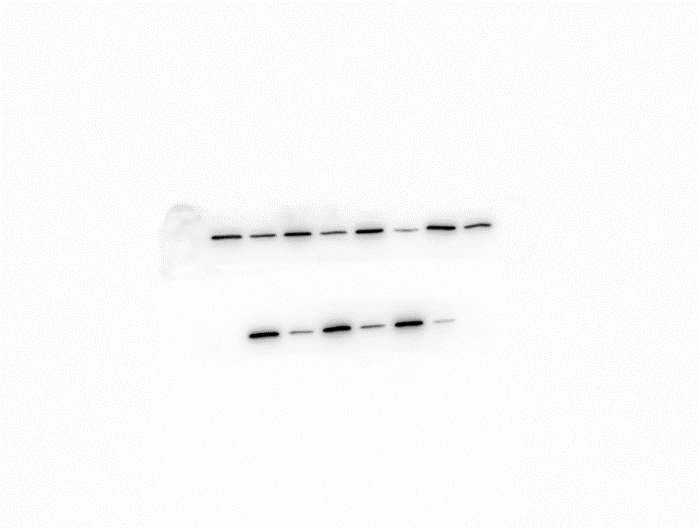 | 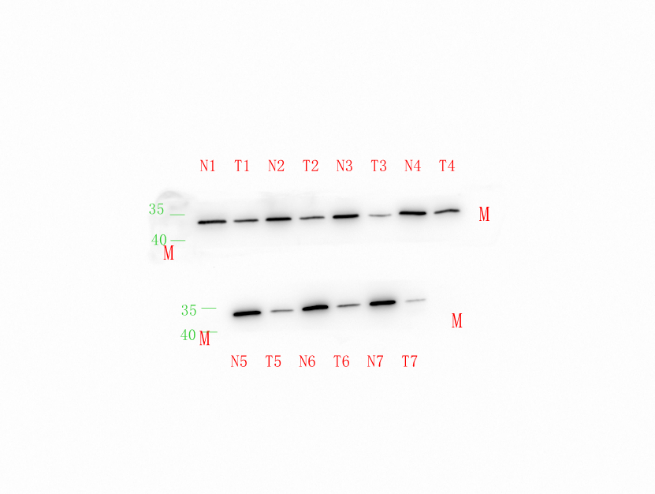 |

1. The original full-length gel/blot bands of LDHB in 7 pairs of ccRCC and adjacent tissues.

| The original full-length gel/blot bands | The marked full-length gel/blot bands |
| --- | --- |
| 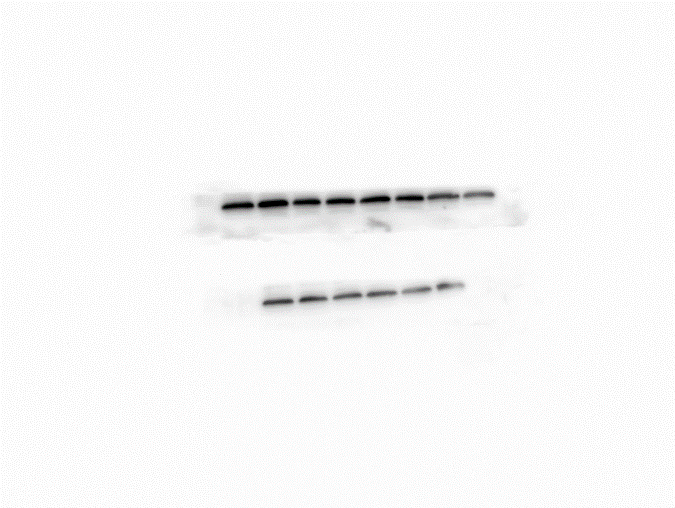 | 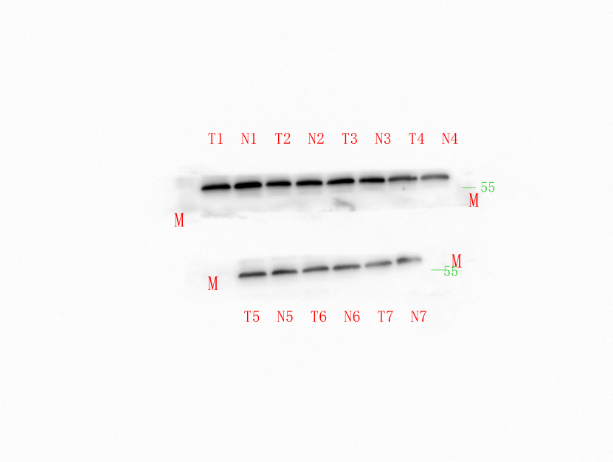 |

1. The original full-length gel/blot bands of Tubulin in 7 pairs of ccRCC and adjacent tissues.
